# Supplementary material for: WordCommentsAnalyzer: A windows software tool for qualitative research
Source: F1000Res. 2018 Sep 4;7:536. Originally published 2018 May 3. [Version 2] doi: 10.12688/f1000research.14819.2 (PMC6137411; doi:10.12688/f1000research.14819.2)
Supplement: Supplementary file 3 [file f1000research-7-17614-s0002.tgz › b2a79496-4ada-47c4-9682-a99ce867836d.zip!/Eight Iranian Health Journals/Journal of Research in Rehabilitation Sciences _21.docx]

© Journal of Research in Rehabilitation Sciences . This work is licensed under CC BY-NC 4.0 (https://creativecommons.org/licenses/by-nc/4.0/).

Volume & Issue: دوره 10، شماره 4: 1393

Title: مقایسه نوسانات پاسچر در وضعیت‌های مختلف ایستادن مردان جوان سالم و مبتلا به زانوی پرانتزی

http://jrrs.mui.ac.ir/index.php/jrrs/article/view/1810

مقدمه: زانوی پرانتزی ریسک فاکتوری برای بروز استئوآرتریت زانو  است. درک تغییر نوسانات پاسچر در وضعیت­های مختلف ایستادن افراد دارای زانوی پرانتزی، بینش خوبی برای پیش­گیری از بروز استئوآرتریت زانو  در این افراد خواهد داد. لذا هدف تحقیق حاضر، مقایسه نوسانات پاسچر در وضعیت‌های مختلف ایستادن مردان جوان سالم و مبتلا به زانوی پرانتزی بود.  مواد و روش­ها: 80 نفر دانشجوی مرد سالم، شامل 40 نفر دارای زانوی پرانتزی و 40 نفر دارای زانوی نرمال در این تحقیق شرکت کردند. ناهنجاری زانوی پرانتزی با استفاده از کولیس و گونیامتر انداز­ه­گیری شد. هر آزمودنی در پنج وضعیت ایستادن روی صفحه نیروسنج قرار گرفت تا تغییرات مرکز فشار او ثبت گردید. برای تجزیه تحلیل داده­ها از نرم افزار Matlab و SPSS و برای مقایسه متغیرهای وابسته مورد نظر از آزمون تحلیل واریانس مختلط(Mixed ANOVA)  در سطح معناداری05/0 P ≤ استفاده شد. نتایج: نوسان پاسچر در وضعیت­های دوپا چشم باز و وضعیت تک پا چشم باز بین دو گروه پرانتزی و نرمال تفاوت معناداری داشت(05/0P ≤) درحالی­که در وضعیت­های دوپا چشم بسته، تک پا چشم بسته و تک پا سربالا تفاوت معناداری مشاهده نشد. بحث: به نظر می‌رسد تغییرات زاویه مفصل زانو در صفحه فرونتال بر نوسانات پاسچر تاثیرگذار باشد. احتمالاً یک دلیل بالاتر بودن خطر آسیب دیدگی و ابتلا به استئوآرتریت زانو  در این افراد افزایش نوسانات مرکز فشار باشد و پیشنهاد می‌شود برای کاهش این خطرات بر تمرینات اصلاحی تمرکز شود.

Title: بررسی روایی و اعتبار نسخه ی فارسی آزمون واژه یابی- ویرایش دوم، در دانش آموزان 8-11 ساله ي طبيعي مدارس شهر اصفهان : فرم متوسط

http://jrrs.mui.ac.ir/index.php/jrrs/article/view/1285

مقدمه: مشکلات واژه یابی در میان دانش آموزان دارای اختلالات زبانی و یادگیری وجود دارد. از رایج ترین راه های ارزیابی واژه یابی،ارزیابی رسمی است که آزمون واژه یابی ویرایش دوم یکی از آنها می باشد. در این مطالعه، به بررسی روایی و اعتبار نسخه ی  فارسی آزمون واژه یابی ویرایش دوم در دانش آموزان 8-11ساله دبستان های شهر اصفهان پرداخته شده است. مواد و روشها: این مطالعه توصیفی- مقطعی بود.  سؤالات آزمون پس از ترجمه و انجام تغییرات لازم بر روی 210دانش آموز 8-11 ساله به صورت تصادفی و با کسب اجازه از والدین ایشان اجرا شد. تحلیل سؤالات با استفاده از نظریه ی سؤال-پاسخ و با وارد نمودن الگوی پاسخ ها به صورت صفر و یک در  نرم افزار BILOG-MG(6) انجام شد. اعتبار آزمون  با استفاده از روش آلفای کرونباخ  بدست آمد و همچنین شاخص های آماری فرم متوسط با استفاده از نرم افزارSPSS(13) محاسبه شد. نتایج : :  میانگین نمرات واژه یابی در سنین 8، 9، 10 و 11ساله به ترتیب برابر با 30/40 ، 51/45 ، 05/48 و 11/54 بدست آمد که نشان دهنده ی افزایش تدریجی میانگین با افزایش سن می باشد(05/0:α و00/0 Pvalue:) . و نیز اعتبار آزمون برابر با 84/. بود .  بحث:قدرت تمييز سوالات آزمون براي دانش آموزان فارسي زبان با قدرت تمييز سوالات آزمون جرمن برابر است . پس آزمون حاضر قابليت خوبي در تمييز بين توانائي هاي واژه یابی مختلف دانش آموزان دارد. همچنين آزمون از لحاظ روانسنجی از اعتبار خوبی برخوردار بود. بدين ترتيب يك آزمون روا و معتبر جهت ارزيابي واژه يابي دانش آموزان فارسي زبان بدست آمد.

Title: مقايسه ي الگوهاي مشارکت کودکان فلج مغزي و کودکان سالم در فعاليت هاي روزمره خارج از مدرسه

http://jrrs.mui.ac.ir/index.php/jrrs/article/view/1157

مقدمه: مشارکت در فعالیت های روزمره دوران کودکی برای رشد سلامت همه کودکان بدون توجه به توانایی یا ناتوانی ضروری می باشد. هدف از مطالعه حاضر بررسی مشارکت کودکان فلج مغزی 7 تا 15 سال در مقایسه با کودکان سالم بوده است. مواد و روش ها: در این مطالعه مقطعی 40 کودک فلج مغزی از مدارس ویژه جسمی- حرکتی و 40 کودک طبیعی همسال آنها از مدارس عادی به روش غیراحتمالی در دسترس انتخاب شدند. مشارکت این کودکان از طریق مصاحبه به وسیله آزمون CAPE ارزیابی شد. داده ها از طریق نرم افزار SPSS17 تجزیه و تحلیل گردید. یافته ها: تنوع و شدت مشارکت در انواع فعالیت ها بجز فعالیت های تفریحی بین دو گروه تفاوت معناداری را نشان داد (p£0.001). کودکان فلج مغزی نسبت به کودکان سالم در فعالیت های مهارتی (0.001>p) و فیزیکی (0.01>p) به تنهایی مشارکت می کردند. کودکان فلج مغزی نسبت به همسالان اکثر فعالیت ها را کمتر در محیط های خارج از خانه انجام می دادند(p£0.001). اثر اصلی جنسیت و اثر متقابل گروه در جنسیت تنها از نظر تنوع انجام فعالیت های فیزیکی معنادار بود (0.05>p).   نتیجه گیری: کودکان سالم در تعداد فعالیت های بیشتری مشارکت داشتند. فلج مغزي مي تواند بر الگوهای  مشارکت کودکان و نوجوانان اثر بگذارد.

Title: تأثیر تمرین تعادلی در آب، ویبریشن کل بدن و بی‌تمرینی بر عملکرد عصبی- عضلانی، تعادل و احتمال به زمین افتادن زنان سالمند

http://jrrs.mui.ac.ir/index.php/jrrs/article/view/1853

مقدمه: اختلال در تعادل از عوامل اثرگذار در افزايش خطر افتادن است که بهبود آن می‌تواند موجب کاهش خطر افتادن گردد. لذا هدف از انجام مطالعه حاضر مقایسه اثر شش هفته تمرین تعادلی در آب و ویبریشن تمام بدن و ترکیبی و بی‌تمرینی متعاقب تمرینات بر عملکرد عصبی- عضلانی، تعادل و احتمال به زمین افتادن زنان سالمند بود. مواد و روش‌ها: 48 نفر از سالمندان در 4 گروه تمرینات تعادلی در آب(13 نفر)، تمرینات ویبریشن تمام بدن(11 نفر)، تمرینات ترکیبی تعادلی و ویبریشن(12نفر) و گروه کنترل(12 نفر) قرار گرفتند. قبل از شروع تمرینات آزمون‌های تعادل ایستا و پویا و تحرک‌پذیری پاسچرال گرفته شد و پس از دو، چهار و شش هفته انجام تمرینات و پس از دو و چهار هفته بی‌تمرینی آزمون‌های مجدد گرفته شد. جهت تجزیه و تحلیل آماری، تحلیل واریانس با اندازه­گیری مکرر، تحلیل واریانس یک‌طرفه انجام شد. نتایج: تعادل ایستا و پویا و تحرک‌پذیری پاسچرال آزمودنی‌ها در گروه‌های تمرین در آب، ویبریشن تمام بدن و گروه ترکیبی پس از انجام تمرینات به‌طورمعنی‌داری بهبود یافت. گروه تمرین ویبریشن و تمرین ترکیبی پس از دو هفته تمرین بهبود معنی‌داری را در میزان تعادل ایستا و پویا و تحرک‌پذیری پاسچرال خود نشان دادند. گروه تمرین در آب پس از 4 هفته تمرین بهبود معنی‌داری را در این متغیرها نشان داد. همچنین پس از دو هفته بی‌تمرینی گروه تمرین ویبریشن و ترکیبی اختلاف معنی‌داری را با پس‌آزمون در میزان تعادل ایستا و پویا و تحرک‌پذیری پاسچرال نشان داد، گروه تمرین در آب پس از 4 هفته بی‌تمرینی اختلاف معنی‌داری را با پس‌آزمون نشان دادند و پس از دو هفته بی‌تمرینی اختلاف معنی‌داری را نشان ندادند. بحث: می‌توان هر سه نوع تمرین را به‌عنوان یک شیوه تمرینی جهت بهبود تعادل ایستا و پویا و نیز بهبود عملکرد عصبی-عضلانی و حرکت‌پذیری پاسچرال برای زنان سالمند سالم پیشنهاد نمود.

Title: بررسی تأثیر بازی‌های کامپیوتری بر سطح توجه کودکان مبتلا به اختلال کم‌توجهی و بیش‌فعالی

http://jrrs.mui.ac.ir/index.php/jrrs/article/view/1772

مقدمه: اختلال کم‌­توجهی و بیش­فعالی الگوی کاهش توجه پایدار، بیش‌فعالی و رفتارهای تکانشی است که معمولا از سنین کودکی شروع شده و تا بزرگسالی ادامه پیدا می­کند. علائم اولیه این بیماری به وسیله مداخلات دارویی درمان می‌شوند، ولی سایر علائم به وسیله ترکیبی از رویکردها قابل درمان است. مطالعات اخیر روی تأثیرگذاری تکنولوژی‌های نرم‌افزاری مثل بازی‌های کامپیوتری، بر حواسپرتی و حرکات اندام‌ها متمرکز شده است. پژوهش حاضر در خصوص بررسی تأثیر بازی‌های کامپیوتری بر سطح توجه کودکان 7 تا 12 سال  مبتلا به اختلال کم‌توجهی و بیش‌فعالی انجام شد. مواد و روش‌ها: در این پژوهش، 18 کودک مبتلا به اختلال کم‌توجهی و بیش‌فعالی مراجعه کننده به بخش روانپزشکی درمانگاه امام رضا (ع) شهر شیراز، به صورت نمونه‌گیری آسان انتخاب شده و به‌صورت نیمه‌تصادفی در دو گروه مداخله و کنترل جای گرفتند. گروه مداخله هر یک 14 جلسه 40 دقیقه‌ای تحت مداخله بازی‌های کامپیوتری قرار گرفتند. سطح توجه هر دو گروه با استفاده از آزمون عملکرد پیوسته (Continuous Performance Test یا CPT) قبل و بعد از مداخله مورد ارزیابی قرار گرفت. داده ها با استفاده از آمار توصیفی، آزمون های من‌ویتنی و ویلکوکسون در نرم افزار آماری SPSS نسخه 18 تحلیل و بررسی گردید. یافته‌ها: در این پژوهش چهار فاکتور سطح توجه در آزمون عملکرد پیوسته یعنی تعداد پاسخ های صحیح، خطاهای حذف، خطاهای ارائه و سرعت عمل مورد بررسی قرار گرفتند. تجزیه و تحلیل نتایج آماری نشان دادند که سرعت عمل در گروه مداخله بعد از مداخله کاهش یافته و از نظر آماری تفاوت معنادار بوده است (P=0/02) اما در بقیه فاکتورها تفاوت معناداری بین گروه‌های تحت مداخله مشاهده نشد. نتیجه گیری: نتایج حاصل از پژوهش حاضر نشان داد که مداخله بازی‌های کامپیوتری تأثیر معناداری بر فاکتورهای سطح توجه آزمون عملکرد پیوسته نداشته است و تنها عاملی که بعد از مداخله بهبود داشته است سرعت عمل کودکان مبتلا به اختلال کم‌توجهی و بیش‌فعالی در گروه مداخله بود.

Title: تاثير تمرين حرکت پرس پا نشسته بر قدرت اکستنشن زانوی سالمندان

http://jrrs.mui.ac.ir/index.php/jrrs/article/view/1370

مقدمه: کاهش قدرت و حجم عضلانی همراه با افزایش سن مسئله شناخته شده ای است که همراه با کاهش قابلیتهای عملکردی می باشد. کاهش قدرت عضله چهارسررانی به عنوان یکی از عوامل پیش بینی کننده افتادن و ترس از افتادن در سالمندان می باشد. با توجه به اهميت قدرت عضله چهارسررانی در سالمندان، هدف از انجام این تحقيق تاثير تمرين حرکت پرس پا نشسته بر قدرت اکستنشن مفصل زانوی سالمندان بود. مواد و روش ها: 24 مرد سالمند (با ميانگين و انحراف استاندارد سني 6±41/61 سال، وزن: 2/6±9/75 کیلوگرم، قد: 2/5±31/173 سانتی متر) به طور تصادفی به دو گروه تمرين قدرتی و کنترل تقسيم شدند. تمرين قدرتي شامل حرکت پرس پا نشسته با دستگاه، با شدت 70% 1RM در يک ست 12 تکراري به مدت هشت هفته و هفته اي دو جلسه در گروه تمرين قدرتی به اجرا درآمد. در اين مدت گروه کنترل به فعاليتهاي روزمره خود مشغول بودند. با استفاده از دستگاه ایزوکینتیک بایودکس سیستم 3 (/sec°60)، قدرت ایزوکینتیک اکستنشن زانو پای برتر و غیر برتر براي هر دو گروه قبل و بعد از دوره تمرینی اندازه گيري شد. يافته ها: پس از هشت هفته تمرین قدرتی، قدرت اکستنشن مفصل زانو پای برتر و غیر برتر در گروه تمرینی به طور معني داري افزايش يافت (به ترتيب 002/0p= و 003/0p=) در حالی که اين تغييرات براي گروه کنترل معني دار نبود (به ترتيب 220/0p= و 168/0p=). نتيجه گيري: هشت هفته دو جلسه ای تمرين قدرتی شامل يک ست 12 تکراري از حرکت پرس پا نشسته با دستگاه با 70% 1RM ممکن است منجر به بهبود قدرت اکستنشن زانوی سالمندان شود.

Title: بررسی و مقایسه تأثیر تغییرات صوتی مرتبط با سن بر کیفیت زندگی سالمندان و میانسالان

http://jrrs.mui.ac.ir/index.php/jrrs/article/view/1708

مقدمه:فرآیند طبیعی مسن شدن با رویدادهای بیولوژیکی همراه است که ساختار و عملکرد بخش‌های مختلف بدن را تغییر می‌دهد.حنجره نیز با افزایش سن از جوانی تا سالمندی،متحمل تغییرات فیزیولوژیک و آناتومیکی گسترده‌ای می‌شود. متناسب با تغییرات آناتومیکی و فیزیولوژیکی حنجره ، ویژگی‌های آکوستیکی و ادراکی صوت نیز درنتیجه­ی تغییرات طبیعی مرتبط با افزایش سن تغییر می‌کند.محدودیت در تولید صوت درنتیجه تغییرات اشاره‌شده می‌تواند تأثیر منفی بر جنبه‌های مختلف زندگی ازجمله حوزه‌های اجتماعی،عملکردی،عاطفی،فیزیکی با افزایش سن داشته باشد.هدف این مطالعه بررسی و مقایسه تأثیر تغییرات صوتی مرتبط با سن بر کیفیت زندگی در افراد میانسال و افراد سالمند بود. مواد و روش‌ها: این مطالعه توصیفی و تحلیلی بود.افراد موردمطالعه شامل 40 فرد میانسال(گروه سنی 60-50سال)و 40 سالمند (گروه سنی 70-60 سال)بودند.پس از بررسی معیارهای ورود و خروج ،بررسی  سلامت شناختی سالمندان با استفاده از  آزمون کوتاه وضعیت ذهنی(MMSE) و ارزیابی کیفیت زندگی بر اساس نمرات شاخص کیفیت زندگی مرتبط با معلولیت صوتی(VHI)صورت گرفت.سپس نمره‌ی کلی VHI و زیرآزمون‌ها برای هر فرد محاسبه شد.نتایج با استفاده از آماره‌های توصیفی و تحلیلی مورد تجزیه‌وتحلیل قرار گرفت.

Title: مقايسه دو نوع فعاليت بدني در آب و خشکي بر فاکتورهاي مرتبط با سلامت زنان سالمند (60 تا 75 ساله)

http://jrrs.mui.ac.ir/index.php/jrrs/article/view/1557

مقدمه: سالمندان يكي از اقشار آسيب پذير هر جامعه اي را تشكيل مي دهند، مهمترين مساله در بهبود سلامت و کيفيت زندگی سالمندان به حفظ استقلال بالای آنها در فعاليت های جسمانی و ادامه زندگی به صورت فعال مربوط می شود. هدف از تحقيق حاضر ، مقايسه تاثير دو نوع فعاليت بدني در آب و خشکي بر فاکتورهاي مرتبط با سلامت (تعادل، انعطاف پذيري، استقامت قلبي – عروقي، قدرت عضلاني، استقامت عضلاني، چابکي) زنان سالمند مي باشد. مواد و روش ها: تعداد 42 زن سالمند واجد شرایط (60 تا75 ساله) انتخاب و در دو گروه تمرين در خشکي (21 نفر) و تمرين در آب (21 نفر) طبقه بندي شدند. تعادل (آزمونY) ، انعطاف پذيري بالاتنه (آزمون کشش پشت) و انعطاف پذيري پايين تنه (آزمون نشستن و رسيدن) ، قدرت عضلاني بالا تنه (آزمون خم کردن بازو) ، قدرت عضلاني پايين تنه (آزمون  برخاستن از صندلي) ،چابکي (آزمون 8 فوت راه رفتن) و براي استقامت قلبي – عروقي (آزمون هاي  6 دقيقه راه رفتن و 2 دقيقه گام برداري) قرار گرفت. نمونه ها بعد از انجام تمرين مورد اندازه گيري قرار گرفتند. داده ها با استفاده از آزمون t مستقل آناليز شدند(05/0P<). يافته ها: نتايج تحقيق نشان داد، که تفاوت معناداري بين دو گروه خشکي و آب در خصوص تعادل چشم بسته، تعادل خلفي جانبي، استقامت قلبي-عروقي (05/0 P>) مشاهد شد، اما در ساير متغيرها (05/0P<)  تفاوت معناداري مشاهده نشد. نتيجه گيري: از يافته هاي اين تحقيق مي توان نتيجه گيري کرد که بکار گيري تمرينات در آب و خشکي منجر به بهبود تمامي فاکتورهاي مرتبط با سلامت سالمندان مي شود، اما تمرينات در خشکي منجر به افزايش استقامت قلبي عروقي و تعادل در سالمندان مي شود. با اين وجود مطالعات بيشتري با حجم نمونه بالاتر و در گروه هاي مختلف سالمندان جهت تعيين تاثير طولاني مدت اين تمرينات پيشنهاد مي شود.

Title: بررسی تأثیر باورهای مذهبی در سلامت عمومی و شادکامی مادران کودکان کم¬توان ذهنی و عادی

http://jrrs.mui.ac.ir/index.php/jrrs/article/view/1544

مقدمه: تولد و حضور كودكي با كم‌تواني ذهني در هر خانواده­ مي‌تواند رويدادي نامطلوب و چالش‌زا تلقي شود. در چنين موقعيتي همه اعضاي خانواده به ويژه مادران در معرض خطر ابتلا به مشكلات مربوط به سلامت عمومی قرار مي‌گیرند. هدف پژوهش حاضر بررسی تأثیر باورهای مذهبی در سلامت عمومی وشادکامی مادران کودکان کم‌توان ذهنی و عادی بود. مواد و روش‌ها: پژوهش حاضر از نوع پژوهش‌های همبستگی بود. نمونة پژوهش شامل 131 مادر کودک کم توان ذهنی بود که به روش تمام­شماری انتخاب شدند و 131 مادر کودک عادی که از طریق همتاسازی با گروه نمونه انتخاب شدند و در نهایت، پرسشنامه­های سلامت عمومی، شادکامی آکسفورد و باورهای اساسی مذهبی برای استخراج داده‌ها مورد استفاده قرار گرفت و داده­های به­دست آمده با استفاده از نرم افزار SPSS و آزمون­های رگرسیون و همبستگی تحلیل شد. یافته‌ها: یافته­های این پژوهش نشان داد بین باورهای مذهبی و سلامت عمومی مادران کودکان کم توان ذهنی و عادی به ترتیب (817/0r=) و (932/0r=) همبستگی معناداری وجود دارد و نمرات باورهای مذهبی مادران کودکان کم­توان ذهنی کمتر از مادران کودکان عادی بود (01/0p<). همچنین بین باورهای مذهبی و شادکامی مادران کودکان کم­توان ذهنی همبستگی مثبت و معنادار وجود داشت (907/r=). مادران دو گروه در میزان باورهای مذهبی، شادکامی و سلامت عمومی با هم تفاوت معنادار داشتند (01/0p<). نتیجه‌‌گیری: بر اساس یافته­های پژوهش می­توان نتیجه گرفت که، مادران کودکان کم‌توان ذهنی نسبت به مادران کودکان عادی در یافتن معنای شادکامی در زندگی، سلامت عمومی و تحکیم اعتقادات مذهبی خود دچار مشکلات بیشتری هستند.

Title: مرور سیستماتیک : رویکرد CO-OP در چه اختلالاتی مورد استفاده قرار گرفته است

http://jrrs.mui.ac.ir/index.php/jrrs/article/view/1351

چكيده مقدمه:CO-OP یک مداخله مراجع­مدار بر پایه شناخت است، که بر توانایی­های فرد جهت رسیدن به اهداف کارکردیش تمرکز دارد، و بر این فرض بنا شده­است که شناخت، نقش مهمی در کسب مهارت­های کارکردی بازی میکند. هدف از این مقاله، مروری سیستماتیک بر سیر مطالعاتی رویکرد CO-OPدر اختلالات مختلف، نتایج و نوع روش­شناسی مطالعات انجام گرفته و شناسایی کاستی­های موجود در این مطالعات میباشد. مواد و روش­ها: از سایت­هایMEDLINE، PUBMED، Wed of knowledge،ScienceDirect، Irandocو با استفاده از کلماتکلیدیرویکرد شناخت­گرا بر عملکردکاری روزمره زندگی، رویکرد شناختی، راهبرد شناختی و ترکیب این کلمات با اختلالات مختلف مقالات استخراج شد که مقالات یافت شده در بازه زمانی 1998 تا 2013 بودند. یافته­ها: نزدیک به 200 مقالهبا استفاده از کلید واژه­ها استخراج گرديدند و با توجه به ملاک­های ورود و خروج، تعداد 27 مقاله انتخاب گردید تا مورد بررسی قرارگیرد. از این تعداد سه مقاله به توصیف اساس و پروتکل CO-OP پرداخته­بودند که در سطح­بندی شواهد مورد بررسی قرار­نگرفتند؛ 17 مقاله دیگر در سطح III و 7 مقاله در سطح V کیفیت قرار گرفتند. تاکنون تاثیر این رویکرد در اختلالات هماهنگی رشدی، آسپرگر، آسیب­مغزی، اوتیسم با عملکرد بالا،اختلال نقص توجه همراه با بیش فعالی، آسیب­مغزی پس از سانحه وسکته­مغزی مورد بررسی قرار­گرفته­است؛ و اثر بخشی آن بر عملکردکاری مبتنی بر حرکت،خودنرخ­دهی عملکرد و رضایت، مشارکت اجتماعی ، استفاده از راهبردها و بهبود آنالیز پویای عملکرد نشان داده شده است. نتیجه­گیری: رویکرد CO-OP اثربخشی مثبتی بر روی اختلالات مورد بررسی داشته­است. با توجه به سطح پایین کیفیت مقالات، این نتایج قابلیت تعمیم را ندارند بنابراین به نظر می­رسد برای افزایش اعتبار اثربخشی این رویکرد در گروه­های مختلف، انجام مطالعاتی که از نظر رتبه­بندی سطح شواهد در سطوح بالا قرار گیرند ضروری است.
